# Neural innervation in adipose tissue, gut, pancreas, and liver

**DOI:** 10.1093/lifemeta/load022

**Published:** 2023-06-06

**Authors:** Mengxue Sun, Yongwen Wan, Mengjie Shi, Zhuo-Xian Meng, Wenwen Zeng

**Affiliations:** Institute for Immunology and School of Medicine, Tsinghua University, and Tsinghua-Peking Center for Life Sciences, Beijing 100084, China; Institute for Immunology and School of Medicine, Tsinghua University, and Tsinghua-Peking Center for Life Sciences, Beijing 100084, China; Department of Pathology and Pathophysiology and Department of Cardiology of the Second Affiliated Hospital, Zhejiang University School of Medicine, Hangzhou, Zhejiang 310058, China; Department of Pathology and Pathophysiology and Department of Cardiology of the Second Affiliated Hospital, Zhejiang University School of Medicine, Hangzhou, Zhejiang 310058, China; Key Laboratory of Disease Proteomics of Zhejiang Province, Zhejiang University School of Medicine, Hangzhou, Zhejiang 310058, China; Institute for Immunology and School of Medicine, Tsinghua University, and Tsinghua-Peking Center for Life Sciences, Beijing 100084, China; Beijing Key Laboratory for Immunological Research on Chronic Diseases, Beijing 100084, China

**Keywords:** neural innervation, peripheral nerves, adipose tissue, pancreas, gut, liver

## Abstract

Efficient communication between the brain and peripheral organs is indispensable for regulating physiological function and maintaining energy homeostasis. The peripheral nervous system (PNS) in vertebrates, consisting of the autonomic and somatic nervous systems, bridges the peripheral organs and the central nervous system (CNS). Metabolic signals are processed by both vagal sensory nerves and somatosensory nerves. The CNS receives sensory inputs via ascending nerves, serves as the coordination and integration center, and subsequently controls internal organs and glands via descending nerves. The autonomic nervous system consists of sympathetic and parasympathetic branches that project peripheral nerves into various anatomical locations to regulate the energy balance. Sympathetic and parasympathetic nerves typically control the reflexive and involuntary functions in organs. In this review article, we outline the innervation of adipose tissue, gut, pancreas, and liver, to illustrate the neurobiological basis of central–peripheral interactions. We emphasize the importance of understanding the functional atlas of neural control of energy metabolism, and more importantly, provide potential avenues for further research in this area.

## Introduction

**Figure 1:**
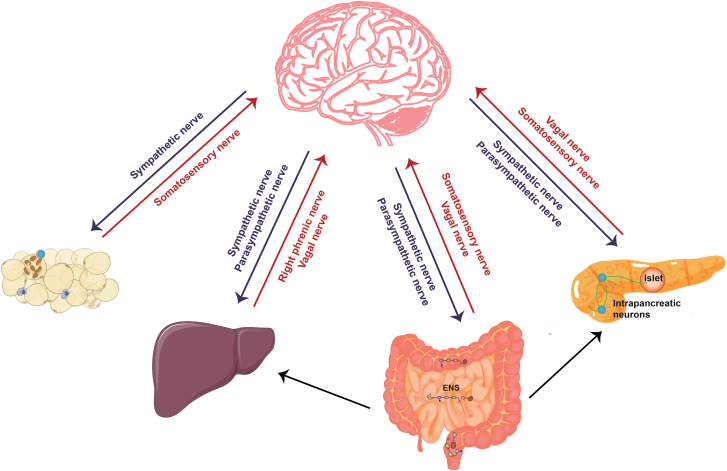
Neuroanatomical distribution in peripheral metabolic organs. The ANS consisting of SNS and PSNS relays information from the CNS to peripheral organs. Somatosensory nerves and vagal sensory nerves deliver peripheral information to the CNS. The right phrenic nerves are mixed nerve bundles containing motor, sympathetic, and somatosensory nerves and innervate the liver. The ENS senses and integrates intrinsic information within the intestine, receives neural input from the CNS, and transmits information to the CNS, sympathetic ganglia, pancreas, and gallbladder. Within the pancreas, in addition to receiving the information from ANS and ENS, the intrapancreatic neurons signal to islets as well as exocrine tissue.

The metabolic organs including adipose tissues, gut, pancreas, and liver are innervated by peripheral nerves to sustain the energy balance [1]. These nerves comprise the peripheral nervous system (PNS), consisting of all nerves branching off from the brain and spinal cord (the central nervous system, or CNS). The PNS can be divided into the autonomic nervous system (ANS) and somatic nervous system: the former includes the sympathetic nervous system (SNS), parasympathetic nervous system (PSNS), and enteric nervous system (ENS), and the latter can be divided into somatosensory and motor parts. Afferent or sensory nerves relay information to the CNS, while efferent or motor nerves transmit impulses away from the CNS. The somatic nervous system carries sensations from the body, such as pain and temperature, and innervates the skeletal muscles under conscious control. The ANS involuntarily regulates blood vessels, smooth muscles, glands, and internal organs. In general, SNS copes with the accelerated increase in physical activity, and PSNS works in the different modes to control the steady state. The ENS is located in the gastrointestinal tract, controlling digestive activity and acting in both CNS-independent and -dependent ways. The vagal nerve is the cranial nerve and a mix of vagal sensory and parasympathetic nerves. Overall, sensory nerves that innervate organ tissues, depending on their origin, can be divided into vagal sensory and somatosensory nerves. CNS integrates information from peripheral visceral glands or organs and then employs SNS and PSNS to innervate them. The various types of nerves work together to control the physiological activities of peripheral organs and eventually unite the body parts in a cohesive entity (Fig. 1) [1].

### Neural innervation of adipose tissues Sympathetic innervation

The adipose tissues are the crucial metabolic organ that regulates systemic energy homeostasis. Based on cellular morphology and functions, the adipose tissues are categorized as brown (BAT), beige, and white adipose tissues (WAT) [2]. Lipids stored in the WAT provide fatty acids for other organs and tissues through lipolysis when needed, and the thermogenic brown and beige fats generate heat for the body in cold conditions. Upon activation by thermogenic or lipolytic signals descending from the brain, the sympathetic nerves expressing tyrosine hydroxylase (TH) release norepinephrine (NE) which acts on adrenergic receptors expressed by the adipocytes and other cells to promote thermogenesis and lipid metabolism [3–5]. Consistent with their various thermogenic capacity, the neural innervations also show heterogeneity among the fat pads. BAT is most densely innervated by the SNS, and NE triggers signal transduction in brown adipocytes to enhance the expression of thermogenic genes and the thermogenic program [6]. To a lesser extent, the beige adipose tissue and subcutaneous WAT are also highly innervated by sympathetic nerves, and sympathetic innervation plays an essential role in the thermogenic activation of the beige fat when the animals are housed in the cold environment [3, 4].

The adipose tissues can undergo thermogenesis, lipolysis, or lipid storage depending on the various stimuli, such as nutrition, stress, or inflammation. Interestingly, the latest study has uncovered that light can activate intrinsically photosensitive retinal ganglion cells (ipRGCs) innervating the hypothalamic supraoptic nucleus (SON), while vasopressin (AVP) and oxytocin (OXT)-expressing neurons in the SON project to the paraventricular nucleus (PVN), then through the GABAergic neurons in the solitary bundle nucleus (NTS) to the rostral raphe pallidus (RPa), a long-range multi-level neural circuit, which eventually inhibits the sympathetic activity to BAT, thereby decreasing glucose tolerance and adaptive thermogenesis in BAT [7]. Studying the engagement of adipose tissues by the wide range of environmental stimuli of this kind is far from being exhausted, particularly at the holistic level. In addition, sympathetic arborization shows a dynamic nature with increased density during cold exposure or cancer-associated cachexia, which leads to alteration of sympathetic control [8–10]. Thus far, it is largely unclear how the external or internal changes may impact sympathetic innervation patterns and thereby affect adipose tissue states.

### Sensory innervation

**Figure 2:**
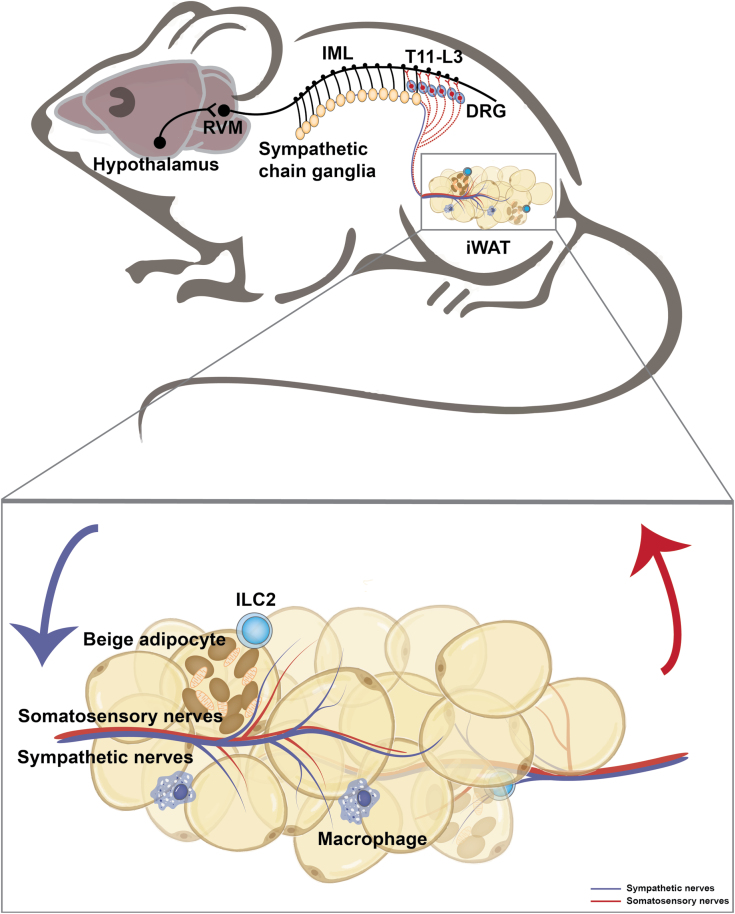
A neuroanatomical model illustrates the sympathetic and somatosensory innervation in the iWAT. The hypothalamic neurons project axons to neurons within the rostral ventromedial medulla (RVM) at the brain stem, which then extend nerves to the sympathetic preganglionic neurons of the intermediolateral nucleus (IML) in the spinal cord. In turn, the preganglionic neurons relay the signal to the sympathetic chain ganglia and further to the iWAT. The somatosensory nerves within the iWAT transmit the peripheral signal to the DRG at the thoracolumbar level (T11-L3).

The presence of somatosensory nerves has been described in the WAT and shown to deliver metabolic signals in adipose tissues to the CNS, such as lipolysis, lipid stores, leptin, etc [11, 12]. However, the precise neural anatomy and physiological functions have not been fully explored [13]. The recent study by Wang *et al*. [14] developed the method termed HYBRiD to directly visualize intact somatosensory innervation from dorsal root ganglia (DRG) to inguinal WAT (iWAT) (Fig. 2). Through nerve tracing and imaging, viral-mediated neuron ablation, and functional characterization, they demonstrated the anatomical basis of adipose sensory innervation, as well as the coordinated function of somatosensory–sympathetic nerve interaction within adipose tissue. By directly injecting a fluorescent protein (FP)-expressing recombinant adeno-associated virus (AAV) into a single DRG (T13/L1) in the thoracolumbar segment and obtaining a global projection from DRG to iWAT, they detected a large number of FP-positive nerve fibers in iWAT. Using the two-color retrograde cholera toxin B subunit (CTB) labeling, they further showed that iWAT and adjacent skin are innervated by two non-overlapping DRG populations. Two main categories of sensory nerves that innervate iWAT are defined: one is a larger bundle traveling along the vasculature, which is TH-negative, and they are parallel to TH sympathetic nerve fibers around but rarely wrap the vessels; the other one is parenchymal innervation, of which about 40% are TH-positive. Next, they examined the function of iWAT-innervating DRG through fat pad-specific denervation. An increase in fat mass and enrichment of multilocular beige adipocytes was observed in the sensory-denervated iWAT. Collectively, the authors found that somatosensory nerves project into iWAT and regulate fat metabolism in a sympathetic innervation-dependent manner. Interestingly, the network of calcitonin gene-related peptide (CGRP)-positive sensory neurons is consistently revealed in WAT in the study by Frei *et al.* [15]. They identified that neuronal protein growth-associated protein 43 (GAP43) can serve as a marker for sensory innervation in WAT [15]. The unveiled sensory innervation in the adipose tissues leads to many immediate and intriguing questions [16]. For instance, how the sensory innervation responds to adipose tissue activity and whether a complex and underappreciated modality is present that mediates the adipose tissue–brain interaction.

### Adipose- and sensory-derived factors

Two modes of communication that convey information about the metabolic state of adipose tissue to the brain have been uncovered and investigated. The first type includes leptin as a key hormone, which is secreted into the circulation by adipose tissue, and then directly acts on the hypothalamus to regulate appetite and systemic metabolism [12]. The second one is sensory innervation in adipose tissue, as studied in-depth by Wang *et al.* [14]. Notably, leptin also functions as a paracrine factor and modulates adipose afferent nerve fibers to convey adipose information to the brain [17, 18]. Besides, other endocrine factors in adipose tissue, such as adipose tissue-derived fatty acids, adipose-derived neurotrophic factors, vascular endothelial growth factors (VEGF), and tumor necrosis factors (TNF), can also modulate local sensory nerve fibers and influence afferent signals back to the CNS [17, 19]. Conversely, secreted factors from sensory neurons, such as CGRP and substance P (SP), may regulate the adipose tissue metabolism as well [17, 20]. It is thereby perceivable that the molecular interaction between the brain and the fat pads mediated by the sensory nerves will be further unfolded with more investigation.

Collectively, the efferent neuronal control of the fat by the brain largely depends on profound sympathetic innervation. The innervation by PSNS has been examined in the adipose tissues, and very few nerve fibers have been observed by far with unresolved roles [11, 13, 21, 22]. On the other side, somatosensory innervation plays an important part in relaying the adipose tissue states to CNS. The emerging findings of crosstalk between sensory and SNS innervation of WAT have underscored the importance of investigating the complete afferent-brain-efferent circuitry in the control of fat metabolism [13].

### Neuroimmune regulation of adipose tissue metabolism

Apart from the neural regulation, the immune microenvironment is a crucial factor in the metabolic activities in the adipose tissues [23, 24]. Various types of immune cells, including macrophages, eosinophils, group 2 innate lymphoid cells (ILC2s), T cells, and B cells, are involved in regulating inflammatory responses, clearing dead cells, and tissue remodeling in metabolic disorders such as obesity [23, 25, 26]. In the case of WAT inflammation, macrophages build up and produce inflammatory cytokines, such as TNF-α and interleukin-6 (IL-6), that disturb the insulin receptors of adipocytes, leading to reduced insulin sensitivity and diabetes [25, 27, 28]. The sympathetic nerve is located in proximity to macrophages, and a particular type of immune cells called sympathetic neuron-associated macrophages (SAMs) express solute carrier family 6 member 2 (SLC6A2), an NE transporter, and monoamine oxidase A (MAOA), a degradation enzyme, that mediate the removal of NE in the WAT [29]. An increase of SAMs was observed in WAT of obese mice, while genetic deletion of *Slc6a2* in SAMs reduces obesity and promotes thermogenesis [29]. Furthermore, during the increase in visceral adiposity associated with aging, neuro-associated macrophages (NAMs) in WAT drive the degradation of catecholamine in an NOD like receptor family pyrin domain containing 3 (NLRP3) inflammasome-dependent manner to blunt lipolysis [30]. Eosinophils, in contrast, have a role in controlling obesity-related WAT inflammation and metabolic diseases, maintaining homeostasis in adipose tissue, and promoting immune health during aging [31, 32]. Cold stimulation-induced fibroblast growth factor 21 (FGF21) acts on adipocytes in an autocrine way and promotes secretion of C-C motif chemokine ligand 11 (CCL11), which recruits eosinophils to subcutaneous WAT and results in macrophage accumulation and beiging [33]. Further, cold stimulation and sympathetic activation induce the release of IL-33 from adipose stromal cells, which acts on ILC2s and promotes IL-5 secretion, leading to accumulation of eosinophils in fat [9]. Eosinophils promote the growth of sympathetic axons and form a feedforward loop between sympathetic nerve activity and type 2 immunity that enhances fat sympathetic innervation and energy expenditure [9]. Thus, neural and immune regulation in adipose tissue is crucial for maintaining adipose tissue function and body energy homeostasis.

## Neural innervation of intestine

The gut represents a vast interface between the host and the ingested food as well as microbes and pathogens. Intestinal nerves are a collection of intrinsic nerves of the ENS and extrinsic nerves of the vagal and somatosensory nerves as well as sympathetic and parasympathetic nerves. The ENS is composed of neurons and glial cells across the entire gastrointestinal tract. ENS also projects to other organs such as the gallbladder, pancreas, and sympathetic ganglia as well as the spinal cord and brain stem [34]. The intestinal nerves function together to control the functions and behaviors of the gastrointestinal tract such as digestion and movements.

### Sensory innervation

Both vagal and spinal afferents respond to gut-derived signals, conveying information about physiological or harmful events to the CNS. The extrinsic sensory nerves project into the gut through the vagal, thoracolumbar, and lumbosacral pathways [34, 35]. According to the specific anatomical ending, the extrinsic sensory neurons of the intestine can be divided into five groups: intraganglionic laminar afferents, mucosal afferents, muscular-mucosal afferents, vagal afferents, and spinal intramuscular afferents [35]. The cell body of vagal sensory neurons is located in nodose ganglia (NG) and their axons fork into two branches, one of which innervates internal organs and the other protrudes into the brainstem [36]. The vagal nerves in the gastrointestinal tract are molecularly heterogeneous and can sense a diverse range of chemical and mechanical stimuli and in turn, induce different metabolic events [37, 38]. For instance, intestinal glucagon-like peptide-1 receptor-positive (GLP1R^+^) vagal afferent detects stomach and intestine stretch [38] and transmits anorexigenic signals to parabrachial nucleus neurons which can control meal termination [39]. G protein-coupled receptor 65-positive (GPR65^+^) vagal sensory neurons target intestinal villi to respond extensively to meal-related stimuli in the intestinal lumen and control gut motility [38]. In addition, stimulation of GPR65^+^ vagal sensory neurons increases hepatic glucose production [39]. Food intake could stimulate the gut mechanoreceptors to effectively and persistently inhibit hunger-promoting agouti-related peptide (AgRP) neurons in the hypothalamus [37]. Neurons in the parabrachial nucleus expressing the *prodynorphin* (*Pdyn*) gene receive mechanosensory signals of the upper digestive tract via the vagus nerve, producing aversive and persistent appetite-suppressing signals that prevent the initiation of eating and drinking [40], which constitutes a negative feedback regulation of eating and drinking behavior to prevent excessive ingestion [41]. Extrinsic sensory afferents derived from DRG contain gut-innervating nociceptors that seem to detect noxious stimuli and disturbances and then mediate sensations such as visceral pain [42–44]. Transient receptor potential vanilloid-1 (TRPV1) nociceptors play a protective role in intestinal tissue through their derivative SP in a microbiome-dependent manner [45]. Nociceptor neurons also prevent colitis by driving mucus production in goblet cells via the CGRP-receptor activity modifying protein-1 (RAMP1) axis [44]. In addition, ingestion of food containing bacterial toxins induces a coordinated defensive response via the gut–brain axis [46]. Vagal sensory neurons expressing 5-hydroxytryptamine receptor 3A (5-HT3R) transmit toxin-related signals from enterochromaffin cells to *preprotachykinin 1* (*Tac1*)-expressing neurons in the dorsal vagal complex (DVC), which drive nausea and retching to eliminate toxins and limit the possibility of re-exposure [46]. In short, gut-innervated extrinsic sensory afferents are complex and precisely regulated, and the underlying intercellular crosstalk and pathophysiological functions are being intensively explored which would yield mechanistic insights in the foreseeable future.

### Sympathetic and parasympathetic innervation

Sympathetic preganglionic neurons innervating the intestine transverse through the thoracic and lumbar spinal cord to the superior mesenteric ganglia, the inferior mesenteric ganglia, or pelvic ganglia [47, 48]. Postganglionic sympathetic fibers enter the intestine and mainly innervate myenteric and submucosal plexuses and blood vessels [48]. Generally, sympathetic nerves of the intestine control blood flow out of the intestine, inhibit intestine motility, and increase the secretion of digestive enzymes [49, 50]. Moreover, SNS innervates gut-associated lymphoid tissue (GALT) and mucosa, which are closely related to intestinal inflammation [51–53]. Its neurotransmitters and modulators, such as NE, neuropeptide Y (NPY), and adenosine triphosphate (ATP), can cause complex anti-inflammatory or pro-inflammatory effects depending on the different concentrations, receptors, and development time of inflammation [52].

Parasympathetic innervation of the gut is provided by the vagal nerve and lumbosacral spinal cord [48]. These vagal efferent nerves originate from the dorsal motor nucleus of the vagus (DMV) within the brainstem and produce two modalities that control gastrointestinal function: an excitatory cholinergic pathway contracts smooth muscle by activating muscarinic cholinergic receptors on the smooth muscle of the gastrointestinal tract, and a non-adrenergic, non-cholinergic (NANC) pathway relaxes smooth muscle by releasing predominantly nitric oxide and/or vasoactive intestinal polypeptide (VIP) [54]. Besides, parasympathetic nerves of the intestine control blood flow into the intestine, promote the intestine motility, and reduce the secretion of digestive enzymes [49, 50]. Vagal efferent nerve stimulation attenuates the systemic inflammatory response caused by endotoxin through a cholinergic anti-inflammatory pathway [55], which is attributed to acetylcholine (ACh) release and the subsequent inhibition of secretion of pro-inflammatory cytokines from macrophage via α-7-nicotinic ACh receptors (α7nAChR) [56]. Moreover, ACh and vagal efferent stimulation can enhance phagocytic potential but reduce the production of pro-inflammatory cytokines via α4/β2nAChR in intestinal macrophages [57].

### Enteric innervation

ENS is a complex network of neurons and glia distributed along the bowel in the myenteric and submucosal plexus. ENS connects intensively with the extrinsic nerves and also contains autonomous neural reflex circuits, mediated by intrinsic sensory neurons (also known as intrinsic primary afferent neurons, IPANs), interneurons, and motor neurons innervating muscles, capable of regulating intestinal function and behavior [34]. ENS sensory neurons respond to nutritional quantity and composition, such as glucose, lipid, and amino acids [58–61]. In turn, ENS plays a variety of roles in gut functions: determining the movement pattern of the gastrointestinal tract, changing local blood flow, controlling the secretion of gastric acid, and interacting with the immune system and endocrine system of the intestine [62]. Studies have also observed a loss of neurons in the gut wall of patients with type 2 diabetes mellitus (T2DM) [63], which suggests that ENS dysfunction is closely correlated with diabetic metabolic disorders. In addition, ENS communicates with organs beyond the intestine, for example, ENS is involved in stimulating the pancreas through the enteropancreatic circuit [64]. During digestion, the intestinal nervous system and enteric nerves regulate motilin release and pancreatic secretion, which is important for postprandial pancreatic enzyme release and gastrointestinal digestive function reflex [65].

**Figure 3:**
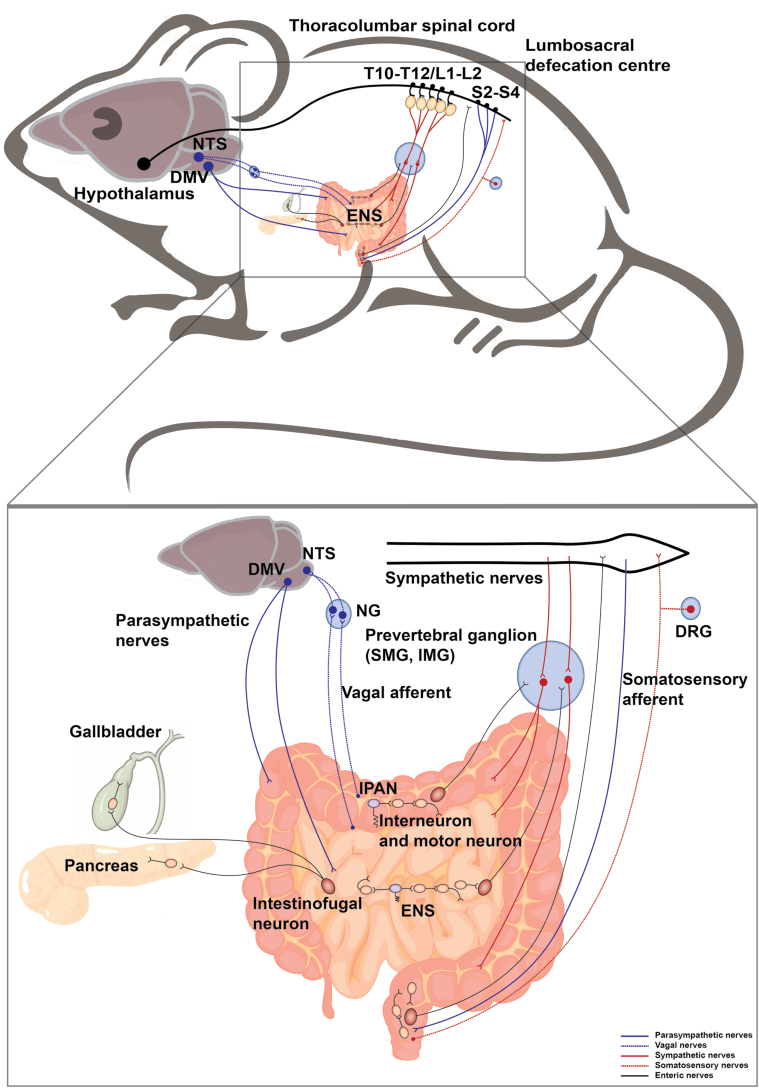
Neural innervation in the intestine. The intestine receives sympathetic and parasympathetic efferent, somatosensory and vagal afferent, and is innervated by ENS. The sympathetic preganglionic neurons reside in the IML in the thoracolumbar spinal cord (T10−T12, L1−L2), which project to the prevertebral ganglia including superior mesenteric ganglion (SMG) and inferior mesenteric ganglion (IMG). The postganglionic sympathetic nerves enter the intestine. The parasympathetic nerves originate from the DMV and lumbosacral spinal cord (S2−S4). The vagal afferent neurons and somatosensory afferent neurons innervate the intestine and their cell bodies are located in NG and DRG, respectively. The intestine contains complete reflex circuits of the ENS (interneurons and motor neurons in yellow, IPANs (sensory neurons) in purple). Pathways from the intestine project outwards from intestinofugal neurons (in red) to the CNS, sympathetic ganglia, pancreas, and gallbladder.

Overall, the afferent information from the intestine to the CNS is conveyed by neurons located in NG and DRG. The efferent nerves employ the conduits of SNS and PSNS motor neurons. ENS organizes intrinsic sensory information in the gastrointestinal tract, controls the intestinal motor output, projects to other gastrointestinal glands, and communicates with CNS (Fig. 3). Together, the extrinsic and intrinsic nerves take part in the sensation of food volume and content, subsequently, the digestion and absorption of nutrients, thereby playing a critical role in the control of metabolic homeostasis.

### Neuroimmune interactions in the intestine

The direct contact with ingested food and the large reservoir of microbes renders the gut nerves at the very frontline exposed to diverse substances. It is thus inevitable that gut neural activities intertwine intensively with the intestinal immune system which would affect the health and disease of the intestine.

The nervous system and innate immune system possess the intrinsic ability to quickly detect and respond to danger signals. Among the immune cells investigated, close communication between intestinal macrophages and neurons has been found, which plays an important role in host defense and tissue protection [66]. Macrophages distributed in the intestinal muscularis externa can regulate the peristaltic activity of the colon by producing bone morphogenetic protein 2 (BMP2) that activates bone morphogenetic protein receptor (BMPR) on enteric neurons. In turn, enteric neurons secrete colony stimulatory factor 1 (CSF1), which sustains macrophages in the intestine [67]. Moreover, neuronal loss induced by intestinal infections is limited by adrenergic signaling in muscularis macrophages. When activated, the gut-innervating sympathetic neurons release NE, which acts on macrophages through the β2 adrenergic receptor (Adrb2). Subsequently, activated macrophages release polyamines to protect enteric neurons from death following enteric infection [68]. Other than macrophages, the tissue-resident ILCs crosstalk with neurons in the gut, which plays an important role in the process of local inflammation and injury. Studies have shown that neuropeptide neuromedin U (NMU) produced by cholinergic neurons promotes the production of IL-5 and IL-13 by ILC2s and promotes the host defense against *Nippostrongylus brasiliensis* infection [69]. In contrast, Adrb2 functions as a negative regulator of the ILC2 response, inhibiting the host defense against helminth infection [70]. Furthermore, VIP produced by VIP^+^ enteric neurons inhibits IL-22 production by ILC3s in a vasoactive intestinal polypeptide receptor 2 (VIPR2)-dependent manner and increases the susceptibility of the host to oral *Citrobacter rodentium* infection [71]. Notably, another study reports that VIP promotes ILC3 expansion and IL-22 production [72]. *VIPR2* deficiency increases the susceptibility to dextran sulfate sodium (DSS)-induced inflammation and intestinal disruption [72]. The seemingly conflicting results could be due to various factors, such as differences in the experimental models or gut microbes and circadian rhythms of ILC3s [43, 73]. Furthermore, the communication between neurons and T lymphocytes in the gut has also been reported. For instance, ACh can directly act on CD4^+^ T cells and promote the production of IL-13 and interferon-γ (IFN-γ) in T cells in an M3 muscarinic ACh receptor (M3R)-dependent manner to enhance host protection during helminth and bacterial infection, respectively [74]. Nevertheless, the neuroimmune regulation mode would benefit from future studies, and an additional layer of complexity can be expected when the microbes-immune crosstalk is being fully considered [75].

## Neural innervation of the pancreas

**Figure 4:**
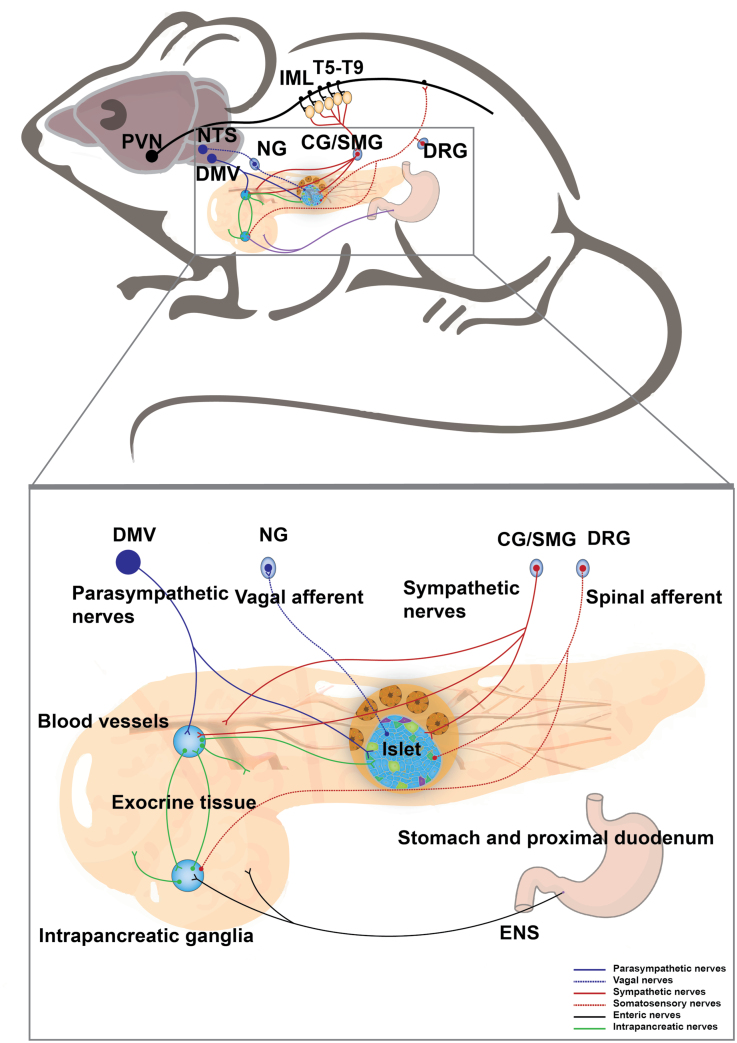
Neural innervation in the pancreas. A schematic shows that the pancreas receives sympathetic and parasympathetic efferent, spinal and vagal afferent, and innervation from the ENS. The neurons in the PVN delay signal to the sympathetic preganglionic neurons of the IML in the thoracic spinal cord (T5−T9), which project to the CG and SMG. The postganglionic sympathetic nerves enter the pancreas and mainly innervate islets and blood vessels. The parasympathetic nerves originate from the DMV and innervate islet and intrapancreatic ganglia. The vagal afferent neurons travel into islets and their cell bodies are located in NG. The spinal sensory afferent fibers with cell bodies in DRG predominantly innervate the exocrine pancreas. Meanwhile, the intrapancreatic ganglia and exocrine pancreas receive inputs from the ENS.

The pancreas performs endocrine and exocrine functions in energy balance, and neuronal control is essential to support pancreatic homeostasis [76]. The pancreas contains two types of cells, the endocrine and exocrine cells. The exocrine cells are mainly composed of acinar cells that synthesize and release digestive enzymes and duct cells that produce chloride and bicarbonate into the small intestine to aid in food digestion and absorption. The endocrine cells are composed of pancreatic islets, also known as the islets of Langerhans, which appear as islands of cells dispersed between the pancreatic acini and constitute up to 2% of the total pancreatic mass. Each islet contains up to a few thousand pancreatic cells and is mainly composed of four types of endocrine cells, secreting glucagon (α-cells), insulin (β-cells), somatostatin (δ-cells), or pancreatic polypeptide (PP-cells) to control systemic glucose, lipid, and protein metabolism [77]. Pancreatic islet β-cell dysfunction is the common cause of both type 1 diabetes mellitus (T1DM) and T2DM [78, 79]. The pancreas is abundantly innervated as described initially by Paul Langerhans [80]. The innervation of the pancreas is controlled by spinal sensory afferent fibers, vagal sensory afferent fibers, sympathetic and parasympathetic fibers, and fibers from the ENS and internal ganglia of the pancreas (Fig. 4).

### Sensory innervation

Spinal cord-derived sensory neurons and vagal sensory neurons transmit tissue information to the CNS and could also release neuropeptides to regulate pancreatic function. The cell bodies of afferent nerves to the spinal cord are located in the thoracic and lumbar DRG, and their fibers travel through the splanchnic nerves and celiac plexus, mainly reaching the exocrine tissue of the pancreas [81]. The pancreatic vagal afferent nerves originate from the NG. Anterograde tracing shows that vagal afferent fibers almost completely innervate the pancreatic islets of rats, supporting their endocrine role [82]. The retrograde AAV tracing reveals that pancreatic vagal sensory neurons project the other branch to NTS in the hindbrain [83]. The vagal sensory nerves express SP, CGRP, and 5-HT3R^83^. As a result, pancreatic infusion of serotonin can activate vagal sensory nerves in the pancreatic islet via 5-HT3R^83^ Nociceptor neurons, existing in the pancreas as an important subset of somatosensory innervation. For instance, selective ablation of TRPV1 sensory neurons enhances glucose-stimulated insulin secretion and glucose clearance without influencing systemic insulin sensitivity or β-cell proliferation in male mice [84]. Capsaicin-mediated ablation of sensory nerves reduces islet inflammation and β-cell stress in the progression of autoimmune diabetes [85]. These results suggest that TRPV1 neurons regulate islet function and systemic glucose homeostasis, and its inactivation may improve glucose metabolism in pathological conditions.

### Sympathetic, parasympathetic, and enteric innervation

The sympathetic preganglionic nerves innervating the pancreas are derived from the thoracic and lumbar spinal cord. They run along the splanchnic nerves to the celiac and superior mesenteric ganglia and then the postganglionic nerve fibers before entering the pancreas to innervate islets, blood vessels, and the exocrine pancreas [86]. Pseudorabies virus (PRV) retrograde tracking has been used to define the islet-brain neuronal map, showing that islets are innervated by efferent circuits emanating from the hypothalamus, and the hypothalamic arcuate nucleus (ARC), ventromedial nucleus (VMN), and lateral hypothalamic area (LHA) significantly overlap with PRV labeling and the glucose-sensing enzyme glucokinase [87]. Recently, a functional transneuronal circuit connecting the hypothalamus to β-cells in mice has been revealed, which springs from a subpopulation of OXT neurons in the PVN (hereafter, PVN^OXT^ neurons), travels through the IML in the spinal cord, and reaches the islets of the endocrine pancreas via sympathetic nerves, where it innervates β-cells [88]. Stimulation of PVN^OXT^ neurons rapidly inhibits β-cells from secreting insulin and causes hyperglycemia, while functional silencing of PVN^OXT^ neurons induces hypoglycemia. Further, glucose deprivation triggers PVN^OXT^ neuronal activity to suppress insulin secretion [88]. Besides, sympathetic nerves that innervate the pancreas promote the release of glucagon [89]. Together, these findings support the crucial role of sympathetic innervations in regulating pancreatic β-cell function and glucose homeostasis.

Parasympathetic innervation of the pancreas originates from the DMV and terminates in the intrapancreatic ganglia and islets. The cell bodies of postganglionic cholinergic nerves are located in the intrapancreatic ganglia, and their axons project to other intrapancreatic ganglia and islets to form a neural network. They primarily release ACh that directly stimulates insulin secretion by β-cells through activation of muscarinic receptors [90]. In addition to the sympathetic and parasympathetic nerves, the pancreas is innervated by the ENS. In rats, serotonergic (5-hydroxytryptamine, 5-HT) fibers from the myenteric plexus of the antrum of the stomach and duodenum project to intrapancreatic ganglia and islets [91].

Together, the neuronal pathways serve as communication routes between the brain and the pancreas (Fig. 4). In-depth studies of the precise brain-pancreatic neural circuits will enrich our understanding of the brain's control of pancreatic physiology and associated pathology.

### Neuroimmune regulation of pancreatic diseases

Pancreatic dysfunction may develop into various diseases, including pancreatitis, diabetes, and tumors. Evidence is emerging that neuroimmune states may be a crucial part of regulating pancreatic homeostasis and pathology [81]. Chemogenetically activated engineered M3R expressed in acinus cells could promote acute pancreatitis, suggesting the pro-inflammatory effect of the neurotransmitter ACh [92]. Differently, the vagal nerves inhibit pancreatic inflammation via the “nicotinic anti-inflammatory pathway” [93], supported by the finding that pancreatitis can be aggravated by vagotomy or treatment with nicotinic receptor antagonists [94]. Nociceptor neurons play a crucial role in pancreatitis-induced pain. Experimental chronic pancreatitis has been associated with augmented expression and currents of TRPV1, whereas TRPV4 and transient receptor potential ankyrin-1 (TRPA1), which respond to penetration and chemical stimuli, are also implicated in mediating pancreatitis-related pain [95, 96]. In addition, TRPV1 and TRPA1 contribute synergistically to pancreatic pain and inflammation [97]. Activation of the nociceptive receptors, depending on its membrane excitability, sets off an action potential that travels to the nociceptive spinal cord terminal, where activation of calcium channels triggers the release of neurotransmitters including SP, CGRP, and brain-derived neurotrophic factor (BDNF), which act upon second-order neurons [98]. These ascending signals are then transmitted to the thalamus, limbic, and cortical structures, leading to the perception and emotional response to pain [98]. Other than this, mast cell degranulation products, such as histamine and 5-HT, may activate nociceptive sensory neurons causing pain [99], whilst neurotrophic factors like nerve growth factor (NGF), artemin, and fractalkine also render nociceptive nerve endings more sensitive [100–102]. On the other hand, patients with chronic pancreatitis and pancreatic cancer show increased pancreatic nerve density and hypertrophy with excruciating pain and inflammatory cell infiltration, indicating the pathological changes of both nerve anatomy and immune microenvironment [103–106].

In addition, pancreatic sympathetic innervation is significantly reduced in chronic pancreatitis and pancreatic cancer [107]. Moreover, the severity of patients with severe pancreatic neuritis, neural invasion by cancer cells, or abdominal pain is associated with noticeably decreased pancreatic sympathetic innervation and cholinergic innervation [107]. However, the effect of sympathetic nerve removal on pancreatic cancer is currently inconclusive. Peripheral chemical sympathectomy appears to promote the development and metastatic spread of a murine model of pancreatic ductal adenocarcinoma (PDAC) and reduce survival [108], while surgery to remove the celiac ganglionic plexus, which cuts off sympathetic efferent inputs from the pancreas, has been shown to prolong survival in mice with established PDAC [109]. Nonetheless, this study also highlights that chronic stress can promote mutated *Kirsten rat sarcoma viral oncogene homologue* (*Kras*)-induced pancreatic tumorigenesis by increasing circulating catecholamines and stimulating Adrb2-dependent pancreatic epithelial growth, as well as upregulating NGF secretion to increase sympathetic innervation and local NE accumulation, generating an Adrb2-neurotrophin feedforward loop to promote pancreatic cancer [109]. Additionally, treatment with isoproterenol has been shown to accelerate pancreatic cancer progression, while β-receptor blocker propranolol acts to increase survival [109, 110]. Thus, the impact of the sympathetic nerve on pancreatic cancer is complex and could be context-dependent, highlighting the need for more in-depth and comprehensive studies.

Longstanding T2DM and pancreatitis have been identified as risk factors for pancreatic cancer [111]. Numerous epidemiological studies have consistently reported an elevated incidence of pancreatic cancer among diabetic individuals, with more than half of PDAC patients also experiencing diabetes-related complications in one study [112, 113]. These findings suggest a bidirectional association between T2DM and PDAC. However, the correlation between pancreatic sympathetic innervation in the development of T2DM, pancreatitis, and pancreatic cancer remains poorly understood.

Within the pancreas of human and mouse T1DM, a loss of sympathetic neurons in islets is found, both early in the disease progression and after the destruction of β-cells [114–116]. In addition, both sympathetic denervation and α1 adrenergic receptor inhibition halt the onset of diabetes in mice [117]. In fact, sympathetic loss in autoimmune diabetes is observed in the pancreatic islets, but not in the exocrine glands [115, 118, 119]. In addition, there is a significant correlation between the degree of selective sympathetic nerve loss and lymphocytic infiltration in the islets in T1DM [119], though it is unclear how T cells may affect nerve structure in the islets [120]. On the other hand, sensory neurons are postulated to “sense” the autoimmunity in T1DM [121]. In the non-obese diabetic (NOD) mouse model of T1DM, pancreatic TRPV1 sensory neurons induce islet inflammation and β-cell stress [85], consistent with the key role of islet innervation in T1DM. In contrast, T2DM is manifested as insulin resistance followed by insufficient insulin production, which causes increased blood glucose levels and is strongly associated with obesity and physical inactivity [122]. Sensory neurons appear to aggravate the T2DM process, as sensory nerve desensitization by resiniferatoxin or capsaicin improves glucose tolerance in obese Zucker rats [123, 124]. In *db/db* mice, an animal model of T2DM, increased pancreatic sympathetic nerve density is identified by three-dimensional panoramic histology [125]. Similarly, an increase in islet nerve density is observed in the pancreas of T2DM patients [126], which suggests that T2DM is associated with significant remodeling of islet nerve input. Given the intricate inter-relationship between the nerves and immune cells in the pancreas, the concept has been evolving, which urges the investigation of diabetes from the integrative neuroimmune-metabolic perspective [17, 127].

## Neural innervation of the liver

**Figure 5:**
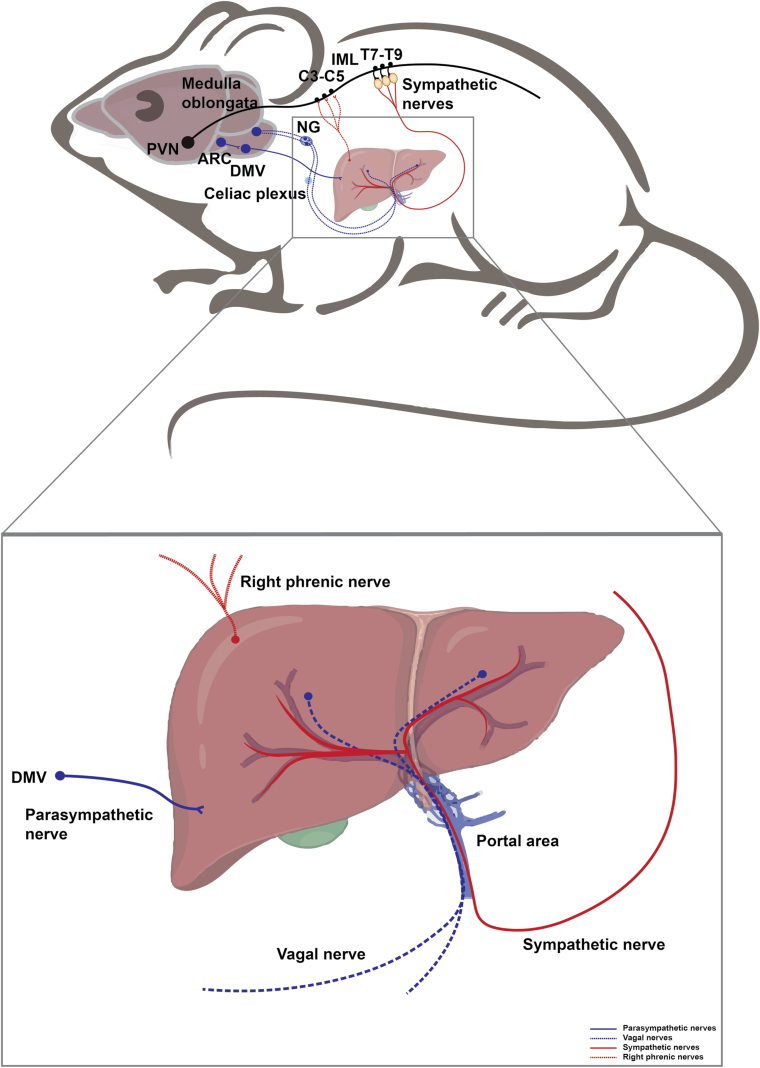
Neural innervation in the liver. The liver is innervated by sympathetic and parasympathetic nerves, vagal nerves, and right phrenic nerves. Hypothalamic PVN neurons descend to activate the sympathetic preganglionic neurons of the IML in the thoracic spinal cord (T7−T9), which further relay the signal to the liver inwards. POMC^+^ neurons in the ARC project to the DMV, which further delivers information into the liver via parasympathetic fibers. Hepatic vagal fibers convey intra-liver information outwards into the medulla oblongata through NG. The liver is also innervated by the right phrenic nerves, which arise from the anterior rami of C3−C5 spinal nerves, consisting of mixed motor, somatosensory nerves, and sympathetic fibers.

The liver is a central metabolic organ in mammals, playing important roles in glucose and lipid metabolism and systemic energy homeostasis. The CNS coordinates the physiological response in the liver via both the SNS and the PSNS [128, 129]. The sympathetic splanchnic nerves innervating the liver originate from neurons in the celiac and superior mesenteric ganglia, which are innervated by preganglionic neurons located in the intermediolateral column of the spinal cord (T7−T12). The parasympathetic nerves innervating the liver originate from preganglionic neurons in the DMV located in the dorsal brainstem [130] (Fig. 5). A large number of autonomic nerve fibers surround the hepatic artery, portal vein, and hepatic lobule to innervate hepatic metabolism [131]. Most of the large nerve bundles adjacent to the portal vein are adrenergic nerve fibers, which are also commonly found around the branches of the hepatic artery but are barely seen near the branches of the portal vein and the bile ducts. In addition, dopaminergic nerve distribution is found around the hepatic artery in humans and rats [132]. Stimulation of sympathetic nerve or adrenergic drug treatment leads to contraction of intrahepatic blood vessels, resulting in a decrease in hepatic blood flow [133, 134]. Stimulation of the parasympathetic nerve or cholinergic drug treatment also produces dilatational changes in the intrahepatic vessels [134]. In the liver, not only the blood vessels are innervated by the autonomic nerves, but also other cell types, such as Kupffer cells, sinusoidal endothelial cells, and hepatic stellate (Ito) cells, are innervated by vagus nerves [135].

### Sensory innervation

At the hilum, autonomic and sensory nerve fibers enter the mammalian liver and form the plexuses along the hepatic artery and portal vein [136, 137]. Changes in visceral osmolality can be detected by sensory neurons that innervate the hepatic portal area (HPA) both directly and indirectly [138]. The sensory fibers in the liver can be activated by extracellular signal-regulated kinase (ERK) signaling, which then sends sensory information to the brain [139, 140]. Additionally, sensory nerves can stimulate the CGRP-RAMP1 pathway, which increases YAP (transcriptional coactivator Yes-associated protein)/TAZ (transcriptional coactivator with PDZ-binding motif) expression and activity, to support liver regeneration [141].

The liver is also capable of degrading toxins from the gastrointestinal tract and systemic circulation [142]. Ethanol produces acetaldehyde during its intermediate metabolism, and acetaldehyde induces neurotoxicity via oxidative stress- and Ca^2+^ imbalance-mediated endoplasmic reticulum stress (ERS) [143]. There is evidence that T-cell activation by staphylococcal enterotoxin B (SEB) strongly stimulates hepatic TNF-α production, and hepatic vagal sensory mechanisms are then activated to transmit toxic signals to the CNS [144–146].

### Neural innervation of liver lipid metabolism

Previous studies using hepatic branch vagotomy (HBV) have demonstrated the important roles of the vagus nerve system in controlling hepatic lipid metabolism. Dysregulation of hepatic lipid and very low-density lipoprotein (VLDL) metabolism plays a causal role in the pathogenesis of obesity and insulin resistance. Hepatic vagal afferent activity can be increased by both portal or jejunal infusion of lipids [147]. Inactivation of the vagal signal by subdiaphragmatic vagotomy mainly regulates the metabolism of VLDL-associated triglyceride (TG) but not LDL-associated or high-density lipoprotein (HDL)-associated TG [148]. Subdiaphragmatic vagotomy-mediated inactivation of the vagal signal inhibits VLDL biogenesis, enhances fecal lipid excretion, and reduces circulating TG, thereby protecting mice from high-fat diet (HFD)-induced hepatic steatosis and hyperlipidemia [148]. Mechanistically, disruption of vagal signaling increases circulating levels of GLP-1, accompanied by significant downregulation of the mRNA and protein expression of sterol regulatory element-binding protein 1c (SREBP-1c), stearoyl-CoA desaturase-1 (SCD-1), and fatty acid synthase (FASN) [148]. The incretin hormone GLP-1 is secreted by intestinal L cells and has been implicated in improving dyslipidemia by reducing lipoprotein production [148]. Changes in temperature also affect lipid metabolism in the liver through the nerves. Cold drives lipid droplet (LD) consumption in the liver through lipophagy, which requires activation of autophagy by proopiomelanocortin (POMC) neurons in the mediobasal hypothalamus (MBH) [149].

Intestinal gluconeogenesis (IGN) is located upstream of and able to signal to the hypothalamus. IGN is capable of modulating hepatic lipid metabolism under the control of the hypothalamus [150, 151]. The activation of IGN prevents hepatic lipid accumulation promoted by a hypercaloric diet and is associated with increased innervation of the liver by TH-expressing neurons, which are widely distributed in sympathetic nerves [151]. Mechanistically, hepatic lipogenesis is controlled by insulin (via SREBP-1c) and by glucose (via carbohydrate-responsive element-binding protein, ChREBP) [152]. A previous study has suggested that the effect of IGN could involve a specific inhibitory action via SREBP-1c of the insulin effect on the lipogenic pathway rather than a regulatory action on the glucose effect via ChREBP [151].

Leptin is an adipokine principally secreted by adipocytes. Leptin signals through binding to leptin receptor in the CNS, transmits to the liver via the vagus nerve, boosts hepatic lipid export, reduces liver *de novo* lipogenesis (DNL), and promotes fatty acid oxidation, thereby protecting the body from ectopic lipid accumulation and development of liver steatosis [153]. Brain leptin’s anti-steatotic effects are generated in the DVC, require hepatic vagal innervation, and are caloric intake independent in rodent models of obesity [153, 154]. Intracerebroventricular (ICV)-delivered leptin exerts its anti-steatotic effect through activation of the signal transducer and activator of transcription-3 (STAT3) signaling in CNS [153]. Recently, in a randomized, placebo-controlled crossover trial, leptin has also been proved to have anti-steatotic effects independent of food intake by stimulating hepatic VLDL-TG export via a brain–vagus–liver axis in humans [155].

However, so far, there is no evidence showing direct innervation of hepatocytes by the vagus nerve. It has been postulated that the glucocorticoid induction of peroxisome proliferator-activated receptor alpha (PPARα)-dependent genes in hepatocytes increases extracellular metabolites in the portal triads, resulting in the activation of hepatic vagal afferent signals, translated by the brain to exert its peripheral effects on insulin sensitivity and glucose and lipid metabolism [156].

### Neural innervation of liver glucose metabolism

It has long been known that the autonomic nervous input to the liver can alter hepatic glucose metabolism [157, 158]. Sympathetic liver activation has been shown to increase liver glucose output by stimulating glycogen breakdown, while parasympathetic liver activation reduces blood glucose levels by promoting glycogen synthesis [159]. Previous studies demonstrate that leptin receptor-expressing POMC neurons normalize blood glucose levels by improving hepatic insulin sensitivity without altering food intake [160]. A recent study shows that a subset of ARC POMC neurons innervates the liver via preganglionic parasympathetic ACh neurons in the DMV. Optogenetic stimulation of the ARC^POMC^ → DMV^ACh^ → liver projection up-regulates the mRNA expression of hepatic gluconeogenic enzymes and elevates blood glucose levels by inhibiting parasympathetic cholinergic outflow to the liver [161]. Interestingly, the effects of these processes were abolished by the treatment with SHU9119, a melanocortin-4 receptor (MC4R) antagonist, or knockdown of the *MC4R* gene in the DMV [161]. Hepatocytes express muscarinic acetylcholine receptors (mAChRs) after receiving cholinergic input from preganglionic parasympathetic cholinergic neurons [161]. Both acute optogenetic stimulation of ARC^POMC^ and NE stimulation can activate the mechanistic target of rapamycin (mTOR) signaling and X-Box binding protein (Xbp1) splicing [162, 163]. Moreover, food perception, transmitted from the hypothalamus to the liver via melanocortin-independent regulation of hepatic sympathetic nerve activity (SNA), promotes mTOR- and Xbp1-dependent coordination of ER homeostasis and liver metabolic functions, such as hepatic glucose production, lipogenesis, and insulin sensitivity [162]. The ARC^POMC^ → MC4R-expressing PVN neuron projection seems to play a minor role in controlling hepatic glucose metabolism [161]. Rather, *MC4R*-expressing PVN neurons serve an important role in the control of body weight, energy expenditure, and food intake, while other types of PVN neurons regulate hepatic glucose production [164]. *MC4R* expression in sympathetic preganglionic neurons can regulate thermogenesis and hepatic glucose production through reciprocal control of sympathetic and parasympathetic preganglionic neuron activity [161, 162]. Interestingly, treatment with MC4R agonists excites preganglionic sympathetic cholinergic neurons in the IML but unexpectedly inhibits preganglionic parasympathetic ACh neurons in the DMV, indicating that melanocortins can oppositely regulate parasympathetic and sympathetic outflow [165].

The vagus nerve has also been shown to regulate food intake and systemic glucose homeostasis by hepatic glycogen content in mice. Previous studies show that protein targeting to glycogen (PTG) over-expression is sufficient to induce hepatic glycogen synthesis and accumulation, protecting the mice from HFD-induced obesity, glucose intolerance, and insulin resistance by decreasing food intake [166]. Intriguingly, HBV reverses the effect of PTG over-expression on food intake, body weight gain, energy expenditure, glucose tolerance, and insulin sensitivity in response to HFD feeding, suggesting an essential role of the hepatic branch of the vagus nerve in the regulation of food intake and glucose homeostasis by liver glycogen [166]. There is an efferent link between the CNS and hepatic glucose production that involves the hepatic branch of the vagus [166]. Hypothalamic lipid metabolism modulates hepatic glucose production by the vagus. Inhibition of lipid oxidation leads to selective activation of neurons in NTS and DMV, which in turn actives ATP-dependent potassium channels (KATP), and decreases the expression of gluconeogenic enzymes via the hepatic branch of the vagus nerve [167].

The gut microbiota regulates tissue physiology, metabolism, and function of both the immune and nervous systems. Notably, a recent study demonstrates that intrinsic enteric-associated neurons (iEANs) can regulate liver gluconeogenesis independently of pancreatic insulin production or intestinal GLP-1 release in a microbiota- and inflammasome-dependent manner [168]. Stimulation or loss-of-function of CART^+^ neurons, a population of autonomous enteric neurons enriched in the ileum and colon, alters blood glucose levels presumably through tissue-specific sympathetic regulation of the pancreas and liver, independently from the CNS [168].

IGN influences the control of glucose and energy homeostasis to exert anti-diabetes and anti-obesity effects [151]. The induction of IGN results in the release of glucose in the portal vein, sensed by a glucose receptor present in the neural system (sodium-glucose co-transporter 3, SGLT3), which sends nerve signals to the hypothalamus to initiate a neural gut–brain axis with benefits for energy homeostasis and reduction of hepatic glucose production [151, 169].

### Neural innervation of both glucose and lipid metabolism

**Figure 6:**
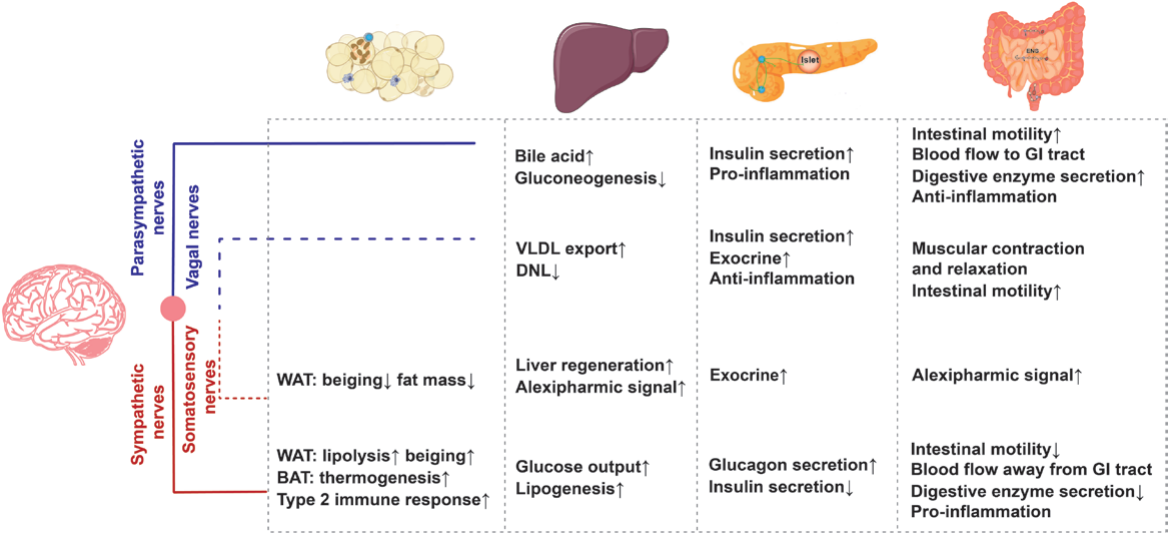
Summary of the functions in peripheral metabolic organs by each neuronal component, including adipose tissue, liver, intestine, and pancreas, illustrated alongside sympathetic nerves (red line), parasympathetic nerves (blue line), vagal nerves (blue dotted line), and somatosensory nerves (red dotted line). WAT, white adipose tissue; BAT, brown adipose tissue; VLDL, very low-density lipoprotein; DNL, *de novo* lipogenesis; GI tract, gastrointestinal tract.

Brain glucose sensing and metabolism have also been implicated in the regulation of food intake [170] and hepatic glucose and lipid metabolism [171, 172], suggesting an important role of the brain–liver axis in the control of systemic energy homeostasis (Fig. 6). It has been reported that elevation of glucose levels in the hypothalamic lowers blood glucose by inhibiting hepatic glucose production in rats, revealing the important role of the brain–liver axis in the regulation of whole-body glucose homeostasis by hypothalamic glucose sensing [172]. Experiments with ICV infusion of glucose, lactate, the LDH inhibitor oxamate, and K_ATP_ channel blocker glibenclamide have demonstrated how glucose in the hypothalamus plays its role in controlling hepatic glucose and lipid metabolism. Glucose is required to be converted to lactate, which then stimulates pyruvate metabolism, triggering the activation of ATP-sensitive potassium channels [171, 172]. In addition, a selective increase of glucose in the brain lowers circulating TG by inhibiting VLDL-TG secretion by the liver [171]. At the mechanistic levels, glucose sensing in the brain inhibits the activity of hepatic SCD-1, which catalyzes the rate-limiting step in the *de novo* synthesis of monounsaturated fatty acid from saturated fatty acids, leading to decreased hepatic assembly and secretion of VLDL particles [171]. The effects of central glucose on liver glucose fluxes are largely due to the significant inhibition of hepatic glucose-6-phosphatase (G6Pase), a key gluconeogenic enzyme [172]. More importantly, these central effects of glucose are rapidly lost in diet-induced obesity in rats, thereby contributing to the development of hyperlipidemia in obesity [171]. During the progression of obesity and its associated metabolic syndrome, chronic inflammation of adipose tissue leads to the abnormal release of adipokines including secreted frizzled-related protein 5 (Sfrp5), a member of the Sfrp family that is decreased in both obesity and type 2 diabetic animals. Interestingly, Sfrp5 was also shown to be expressed and downregulated in the brain of obese and diabetic animals, suggesting a central role of Sfrp5 in systemic metabolic control [173]. Remarkably, a recent study revealed an unexpected role of an Sfrp5-dependent brain-hepatic vagus neurocircuitry in controlling whole-body glucose and lipid metabolism. ICV-delivered Sfrp5 activates the insulin receptor-phosphoinositide 3-kinase (PI3K)—serine/threonine protein kinase B (Akt) pathway in the hypothalamic MBH, and facilitates the *N*-methyl-d-aspartate (NMDA) receptors-mediated neurotransmission of DVC to the hepatic vagus nerve to suppress hepatic glucose production and VLDL-TG secretion [173].

### Neural innervation of hepatic cells except for hepatocytes

Besides hepatocytes, the liver also has multiple other cell types such as Kupffer cells, endothelial cells, sinusoidal endothelial cells, and Ito cells. Interestingly, a recent study reveals that a novel branch of anti-inflammatory circuits of the vagus nerve increases the phagocytic activity by Kupffer cells in addition to its classical effects on the regulation of cytokine production [174]. The activation of the vagus nerve increases the expression of choline acetyltransferase (ChAT) in Kupffer cells, leading to increased acetylcholine synthesis [174]. The efferent cholinergic arm of the vagus in the liver parenchyma can regulate Kupffer cell phagocytosis upon receiving cholinergic signals [174]. Further investigation shows that celiac ganglion (CG) and adrenal gland have an inhibitory effect on the bacterial phagocytosis by Kupffer cells in the liver [174]. The vagus nerve inhibits hepatic IL-6/STAT3 signaling via α7nAchR on Kupffer cells, and central insulin action activates hepatic IL-6/STAT3 signaling by suppressing vagal activity [175]. The inhibition of hepatic vagus activity decreases the content of ChAT in Kupffer cells and suppresses the gene expression of hepatic gluconeogenic enzymes [175]. Studies have shown that the sympathetic nerve induces Ito cells to release prostaglandin via the neurotransmitter NE and the co-transmitter ATP, which activates glycogenolysis in hepatocytes [176].

## Perspective

The neuronal sensation and control in the metabolic organs are increasingly appreciated as a crucial component affecting metabolic activities. Currently, it is recognized that adipose tissue is intensively regulated by sympathetic nerves, while other types of neural innervation are less explored, among which the regulatory role of somatosensory nerves starts to be revealed. Thus far, we still lack detailed molecular characterization of nerve endings in the various layers of the gut, and how they transmit various signals to the spinal cord or brain region is largely unclear. Furthermore, the communication mode and cooperation of ENS with other nervous systems require much accurate anatomical evidence. The distribution of intrapancreatic ganglia between acinar, duct, and islet, as well as ENS and the innervation of extrinsic nerves along the blood vessels into the pancreas, undoubtedly makes it difficult to unravel the anatomical structure of axon bundles and terminals as well as the specific cell types innervated. The liver is a multifunctional metabolic organ, and the influence of hormones such as leptin and insulin cannot be completely ruled out when studying the effects of innervation on hepatic metabolism.

Recent advances in the experimental approaches to optogenetics, viral tools, multi-omics, pharmacology, sequencing, and imaging techniques, together with animal models and human subjects, have been invaluable for unraveling the innervation pattern and functional networks in various organs. In parallel, the functional effects of neuronal populations in the CNS have also been greatly magnified. These findings have highlighted the emerging need and power to better understand inter-organ communications, especially the neuronal pathways between the brain and metabolic organs. The accumulated knowledge will shed light on our therapeutic adventure in altering the energy balance and treating metabolic disorders, such as obesity, diabetes, and cardiovascular diseases.
